# Association between Ngb polymorphisms and ischemic stroke in the Southern Chinese Han population

**DOI:** 10.1186/1471-2350-9-110

**Published:** 2008-12-16

**Authors:** Yi Lin, Ling Fang, Xie-Hua Xue, Shen-Xing Murong, Ning Wang, Zhi-Ying Wu

**Affiliations:** 1 Department of Neurology and Institute of Neurology, First Affiliated Hospital, Fujian Medical University, 20 Chazhong Road, Fuzhou 350005, PR China; 2 Department of Neurology and Institute of Neurology, Huashan Hospital, Shanghai Medical College, Fudan University, 12 Wulumuqi Zhong Road, Shanghai 200040, PR China; 3 Center of Neuroscience, Fujian Medical University, 20 Chazhong Road, Fuzhou 350005, PR China; 4 Institutes of Brain Science and State Key Laboratory of Medical Neurobiology, Shanghai Medical College, Fudan University, 138 Yixueyuan Road, Shanghai 200040, PR China

## Abstract

**Background:**

*Neuroglobin *(*Ngb*), one of novel members of the globin superfamily, is expressed predominantly in brain neurons, and appears to modulate hypoxic-ischemic insults. The mechanisms underlying *Ngb*-mediated neuronal protection are still unclear. For it is one of the candidate protective factors for ischemic stroke, we conducted a case-control study to clarify the association of *Ngb *polymorphisms with ischemic stroke in the Southern Chinese Han population.

**Methods:**

355 cases and 158 controls were recruited. With brain imaging, cases were subdivided into large-artery atherosclerosis (LVD) and small-vessel occlusion (SVD) stroke. PCR amplified all the four exons of *Ngb *and flanking intron sequence for each exon. Genotyping for *Ngb *was achieved by direct sequencing and mismatched PCR-RFLP. Polymorphisms were studied both individually and as haplotypes in each group and subgroup which subdivided according to gender or age.

**Results:**

Two intronic polymorphisms 89+104 c>t and 322-110 (6a)>5a were identified. The allele frequency of 89+104 t was decreased in stroke cases. The protective effect seems to be more pronounced in subgroups of female patients and age > 60 years. Also, we have confirmed decreased LDL-C level and reduced hypertension and hypercholesterolemia in 89+104 t allele carriers. In contrast, the 322-110 (6a)>5a genotype distribution was similar between cases and controls. However, the haplotype 89+104 c>t/322-110 (6a)>5a was related with LVD and SVD stroke. The haplotype c-5a was more frequent in both LVD and SVD groups while t-6a was more frequent in controls.

**Conclusion:**

Ngb polymorphism 89+104 t had protective effects on LVD and SVD in the Southern Chinese Han population. A "hitchhiking" effect was observed for the 89+104 t/322-110 (6a) genotype combination especially for LVD.

## Background

*Neuroglobin *(Ngb, LocusID: 58157; GenBank: DQ008010) is a newly discovered globular heme protein in the vertebrate brain that displays a high affinity for oxygen like *myoglobin *(*Mb*) and *hemoglobin *(*Hb*) [1]. *Ngb *is preferentially expressed in the neurons, as well as some endocrine tissues [1,2]. The highest *Ngb *concentration has been found in the retina, which is also the highest O_2_-consuming organ of the body [3].

*Ngb *is hypoxia-inducible in cultures of cerebral cortical neurons [4] and some neuronal cell lines, such as PC12, immortalized rat hippocampal neuron (HN33) [5,6] and hybrid dorsal root ganglia neuroblastoma cell (ND15) [7]. Prolonged or sustained hypoxia increased *Ngb *expression in vitro [5] or in vivo [8]. The increasing magnitudes of Ngb mRNA levels, ranging from 150% to 500%, can be explained by various experimental conditions or cell lines [6,8,9]. For example, Ngb mRNA was time-dependently upregulated during the first 8 h in a constant hypoxic environment of 1% O_2_, while no significant upregulation occured before 48 h in 3% O_2 _[6]. However, there were contradictory data as to whether Ngb was upregulated under hypoxic conditions in brain [10,11]. Several factors, namely species, brain region, severity and duration of hypoxia, and the pattern of hypoxic exposures, could have accounted for such disparate findings [9]. Cerebral ischemia is another stimulus to *Ngb *expression, although findings vary depending on the model used and the experiment designed [12]. Focal cerebral ischemia is clearly associated with *Ngb *induction, especially in the penumbra [4]. Transient global forebrain ischemia increases *Ngb *expression in the gerbil cerebral cortex and serum, but not in the hippocampus [13].

*Ngb *appears to show the neuroprotective potential following hypoxic and ischemic insults [13-15]. In cultured cortical neurons, antisense-mediated down-regulation of *Ngb *decreases cell viability under hypoxia, whereas additional *Ngb *improves HN33 cells survival [4]. After transfected with *Ngb*-expressing plasmid, human neuroblastoma cells (SH-SY5Y) are resistant to oxidative injury induced by H_2_O_2 _[16]. Intracellular delivery of *Ngb *by human immunodeficiency virus-1 transactivator of transcription (TAT) protein transduction domain fails to rescue rat retinal ganglion (RGC-5) or SH-SY5Y cells from combined oxygen and glucose deprivation [17], whereas in another case, *Ngb*-TAT-treated cultured rat cortical neurons shows reduced sensitivity to hypoxia [18]. Administration of anti-sense oligodeoxynucleotides directed against *Ngb *exacerbates experimental stroke in vivo, while intracerebral administration of *Ngb *with an adeno-associated virus vector reduces the focal cerebral infarct size indeed [15]. Furthermore, overexpression of *Ngb *in a transgenic mouse model reduces cerebral infarct size following middle cerebral artery occlusion (MCAO) [14,19].

However, the mechanisms underlying *Ngb*-mediated neuronal protection during hypoxic/ischemic stress remain largely unknown. It seems to us unlikely that *Ngb *is primarily an O_2 _transporter, because of its fairly low average tissue concentration (approximately micromolar) compared with Mb (0.2 mM or so) in muscles [20]. Several proposed mechanisms include *Ngb *acting as an oxygen sensor and storage molecule [21,22], operating as a guanine nucleotide dissociation inhibitor and then increasing levels of free Gβγ [23], interacting with neuronal membrane proteins including Na-K-ATPase [24] and flotillin-1 [25], and even acting as an intracellular ROS (reactive oxygen species)/RNS (reactive nitrogen species) scavenger [20,26] via reduction reactions leading to ferric-*Ngb *conversion to ferrous-*Ngb *by endogenous reducing enzyme systems [27]. *Ngb *not only decreases oxidative stress induced ROS/RNS overproduction and lipid peroxidation [4,14,28], but also attenuates subsequent mitochondrial dysfunction, apoptosis, and cell death [30,29]. Its antioxidant properties were consistent with the protective role against oxidative stress-induced injury [31]. Recently met *NGB *(ferric-*NGB*) was found acting not only as scavenger of reactive species, but also as a target of the self-generated reactive species [32].

Whatever its function and mechanism is, it can be summarized that the majority of studies agrees that *Ngb *is beneficial for neuronal survival during hypoxic/ischemic stress. Based on aforementioned considerations, we hypothesize that *Ngb *may be one of the candidate genes associated with ischemic stroke. Human Ngb, located on chromosome 14q24, has a 3 intron/4 exon structure [1,33]. The aim of this study is to investigate the association between *Ngb *polymorphisms or genotypes and ischemic stroke in the Southern Chinese Han population, which is the first allele association study on human *Ngb *and stroke.

## Methods

### Subjects

Patients undergoing cranial computed tomography (CT) and magnetic resonance imaging (MRI) and diagnosed as ischemic stroke according to World Health Organization (WHO) criteria were prospectively recruited between September 1, 2004 and February 28, 2007. Cases with a history of malignant disorders, infections, cardiovascular diseases and peripheral vascular diseases, atrial fibrillation, cerebrovascular malformations, brain tumors and traumatic cerebrovascular diseases were excluded. According to the clinical features and the results of diagnostic workup, the following causes of stroke were diagnosed based on TOAST criteria [34]: LVD stroke, SVD stroke, cardioembolic (CE) stroke, and stroke of other determined or undetermined etiology. Only patients with LVD stroke and SVD stroke were included for further analysis. Among them, 221 patients had LVD stroke (160 males and 61 females) and the mean age at onset was 66.61 ± 10.47 years (range: 41–88 years), 134 patients had SVD stroke (76 males and 58 females) and the mean age at onset was 66.96 ± 7.62 years (range: 51–84 years). 158 unrelated age- and sex-matched controls (103 males and 55 females, mean age: 65.08 ± 10.44 years, range: 48–83 years) free of clinically detectable cardiovascular or cerebrovascular disease and positive family history of cerebrovascular disease were recruited from persons undergoing annual medical examination. All subjects came from the Southern Chinese Han population. The study was approved by the local ethics committee, and informed consent was obtained from all participants. Demographic data were collected from stroke patients and controls. Hypertension was defined as repeatedly elevated blood pressure (mean of 3 measurements) exceeding 140/90 mmHg or the use of antihypertensive drugs. Diabetes mellitus is characterized by recurrent or persistent hyperglycemia, and is diagnosed by demonstrating any one of the following three:(1) fasting plasma glucose level at or above 126 mg/dL (7.0 mmol/L), (2) plasma glucose at or above 200 mg/dL (11.1 mmol/L) two hours after a 75 g oral glucose load as in a glucose tolerance test, (3) random plasma glucose at or above 200 mg/dL (11.1 mmol/L). Cigarette smoking is defined as having smoked at least 1 cigarette per day for 1 year or more, and former smokers whose smoke cessation more than five years are not included. Alcohol consumption is defined as drinking alcohol at least 12 times during the last year. [35]

### Genotyping

**Figure 1:**
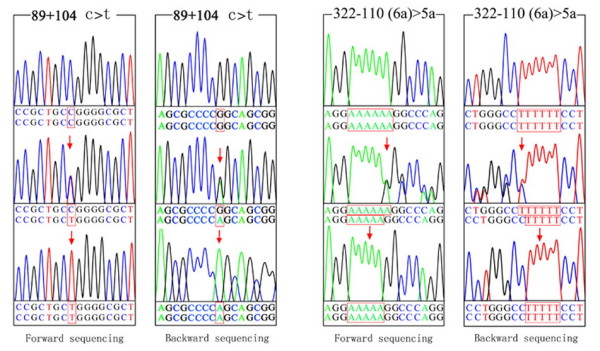
**Chromatograms of the polymorphisms 89+104 c>t and 322-110 (6a)>5a identified in the *Ngb *gene**. Normal sequences are shown in the upper of each box, and the corresponding polymorphisms are shown in middle and down of each box.

**Figure 2:**
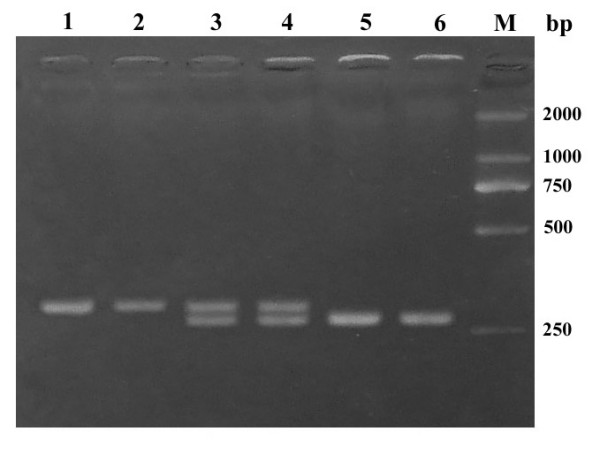
**Mismatched PCR-RFLP analysis of the 89+104 c>t polymorphism**. KpnI recognition site was created in the PCR product by means of mismatched primers KpnI*Ngb*. After cleavage with KpnI, the cc individuals (lane 5 and 6) carried a 260 bp fragment, the tt individuals (lane 1 and 2) carried a 284 bp fragment, and the ct individuals (lane 3 and 4) carried both 260 bp and 284 bp fragments. The size marker (M) is D2000 ranging from 100 bp to 2000 bp.

Genomic DNA was extracted from peripheral blood with QIAamp DNA Blood Minikit (QIAGEN GmbH, Hilden, Germany). Primers used for PCR are illustrated in additional file 1 (Suppl. Table 1. Primers of Ngb and conditions of PCR). PCR amplified all the four exons of Ngb and 95–303 base pairs of the flanking intron sequence for each exon. DNA sequencing was performed with an ABI Prism 3730 genetic analyzer (Applied Biosystems Inc., Foster City, Calif.) by using an ABI dye terminator cycle sequencing kit (Fig. 1). The mismatched PCR-RFLP (primers were showed in Suppl. Table 1.) was used for screening the detected polymorphisms. Ten microlitres of PCR product were digested with the appropriate restriction enzyme according to the manufacturer's recommendations (New England Biolabs, Beverly, MA, USA) and followed by a 2.5% agarose gel electrophoresis (Fig. 2). The D2000 marker (Tianwei Inc, Beijing, China) ranging from 100 bp to 2000 bp was used as the size marker.

### Statistical Analysis

Data on quantitative characteristics are expressed as means ± SD. Data on qualitative characteristics are expressed as percent values or absolute numbers, as indicated. Differences in demographic characteristics and vascular risk factors between patients and controls were initially compared by univariate analysis using Student's t test (for 2 groups) or ANOVA (for 3 groups) for continuous variables and the χ^2 ^test for all categorical variables. Tests for Hardy-Weinberg equilibrium were conducted using χ^2 ^tests. Genotypes and allele frequencies were compared by χ^2 ^analysis or Fisher's exact test. Multivariate logistic regression analysis was used to determine the influence of Ngb polymorphisms on disease risk, controlling potential confounding risk variables including age, sex, and other conventional risk factors. A forward stepwise (Likelihood Ratio) procedure was used for multivariable analysis. The associations of haplotypes comprising *Ngb polymorphisms *with ischemic stroke were estimated using SHEsis Software [36]. Data were analyzed with the SPSS 13.0 package (SPSS Inc) and results were considered statistically significant at P < 0.01 using a 2-tailed test. The two intronic SNPs, 89+104 c>t (rs4903565) and 322-110 (6a)>5a (rs28909968) of Neuroglobin were identified. Genotyping observations were performed blind by different investigators. The post hoc power analysis was done by PASS2005 software.

## Results

### Subject Characteristics

Demographic data and risk factor profiles of patients and controls are presented in additional file 2 (Suppl. Table 2. Clinical and biochemical characteristics of three groups). Concerning the gender, BMI, alcohol intake and smoking, there were no significant differences between cases and controls. However, some risk factors did differ. For example, hypertension and diabetes were more common than controls in LVD, but only hypertension was more common in SVD patients. LDL-C, TC and TG levels were significantly higher in LVD than in controls, while only LDL-C level was higher in SVD. HDL-C level was lower in stroke patients especially in LVD patients. Mean age and gender composition did not differ between LVD or SVD patients.

### Association with Ngb Genotypes and Alleles

**Figure 3:**
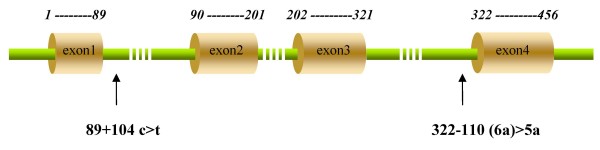
**The positions of the two SNPs in the Ngb gene**. '*1 -89, 90 -201, 202 -321, 322 -456*' show the positions of each exon in the human Ngb cDNA sequence. The intronic SNP positions in the human Ngb gene are indicated by arrows.

Two intronic SNPs, 89+104 c>t (rs4903565) and 322-110 (6a)>5a (rs28909968) of *Neuroglobin*, were identified in this study (Fig. 1 and 3). Both polymorphisms were in Hardy-Weinberg equilibrium for the total group and each group separately. Among all the 513 subjects, genotyping of 89+104 c>t for 120 LVD and 120 SVD patients was all done by direct sequencing. The miamatched PCR-RFLP was used for the remained 273 subjects. Genotypes and allele frequency distributions are presented in Table 1. In the control group, the frequencies of the cc, ct and tt genotypes for 89+104 c>t were 0.146, 0.538, and 0.316, respectively. The frequency of the tt genotype and the frequency of t allele was significantly lower in the LVD and SVD group than in the control group (P < 0.01). When the sexes were analyzed separately, men with SVD did not have significantly lower t allele frequency than those without (P = 0.036); while men with LVD and women with LVD or SVD had a significant lower t allele frequency (P < 0.01). After separated into >60 and ≤ 60 years subgroups, the tendency to t allele frequency decreases can be observed by comparing the control group to patients with age >60 years (P < 0.01), although patients with age ≤ 60 years had a lower t allele frequency too, which was not significant (P = 0.011 and 0.030 for LVD and SVD). Genotyping of 322-110 (6a)>5a was all done by direct sequencing. The 322-110 (6a)>5a genotype distribution was similar in patients with LVD or SVD stroke when compared with controls, which may be due to lack of power (power = 0.0794 and 0.2359 for LVD and SVD respectively) or there is no association in fact.

**Table 1:** Genotype and allele frequency distributions of *Ngb *polymorphisms

***Ngb *polymorphism**	**All**			**Male**			**Female**		
			
	Controls n (%)	LVD n (%)	SVD n (%)	Controls n (%)	LVD n (%)	SVD n (%)	Controls n (%)	LVD n (%)	SVD n (%)
89+104 c>t									
cc	23(14.6)	92(41.6)	52(38.8)	14(13.6)	67(41.9)	27(35.5)	9(16.4)	25(41.0)	25(43.1)
ct	85(53.8)	97(43.9)	56(41.8)	67(65.0)	66(41.3)	34(44.7)	18(32.7)	31(50.8)	22(37.9)
tt	50(31.6)	32(14.5) **	26(19.4) **	22(21.4)	27(16.8) **	15(19.8)**	28(50.9)	5(8.2)**	11(19.0)**
c	131(41.5)	281(63.6)	160(59.7)	95(46.1)	200(62.5)	88(57.9)	36(32.7)	81(66.4)	72(62.1)
t	185(58.5)	161(36.4)**	108(40.3)**	111(53.9)	120(37.5)**	64(42.1)*	74(67.3)	41(33.6)**	44(37.9)**
H-W	0.173	0.437	0.128						
322-110 (6a)>5a									
6a6a	39(24.7)	53(24.0)	32(23.9)	25(24.3)	39(24.4)	18(23.7)	14(25.5)	14(23.0)	14(24.1)
6a5a	88(55.7)	118(53.4)	63(47.0)	58(56.3)	81(50.6)	38(50.0)	30(54.5)	37(60.7)	25(43.1)
5a5a	31(19.6)	50(22.6)	39(29.1)	20(19.4)	40(25.0)	20(26.3)	11(20.0)	10(16.4)	19(32.8)
6a	166(52.5)	224(50.7)	127(47.4)	108(52.4)	159(49.7)	74(48.7)	58(52.7)	65(53.3)	53(45.7)
5a	150(47.5)	218(49.3)	141(52.6)	98(47.6)	161(50.3)	78(51.3)	52(47.3)	57(46.7)	63(54.3)
H-W	0.142	0.312	0.508						

***Ngb *polymorphism**	**≤ 60 years**			**>60years**					
				
	Controls n (%)	LVD n (%)	SVD n (%)	Controls n (%)	LVD n (%)	SVD n (%)			

89+104 c>t									
cc	13(18.8)	26(38.2)	15(41.7)	10(11.2)	66(43.1)	37(37.8)			
ct	37(53.6)	32(47.1)	15(41.7)	48(53.9)	65(42.5)	41(41.8)			
tt	19(27.5)	10(14.7)*	6(16.6)*	31(34.9)	22(14.4)**	20(20.4)**			
c	63(45.7)	84(61.8)	45(62.5)	68(38.2)	197(64.4)	115(58.7)			
t	75(54.3)	52(38.2)*	27(37.5)*	110(61.8)	109(35.6)**	81(41.3)**			
322-110 (6a)>5a									
6a6a	18(26.1)	16(23.5)	15(41.7)	21(23.6)	37(24.2)	17(17.3)			
6a5a	36(52.2)	40(58.8)	14(38.9)	52(58.4)	78(51.0)	49(50.0)			
5a5a	15(21.7)	12(17.6)	7(19.4)	16(18.0)	38(24.8)	32(32.7)			
6a	72(52.2)	72(52.9)	44(61.1)	94(52.8)	152(49.7)	83(42.3)			
5a	66(47.8)	64(47.1)	28(38.9)	84(47.2)	154(50.3)	113(57.7)			

Compared with control group: *P < 0.05, **P < 0.01

H-W indicates Pearson's P value for χ^2 ^test of Hardy-Weinberg equilibrium.

Odds Ratios for associations between *Ngb *polymorphisms and LVD or SVD phenotype were showed in Table 2, Table 3 and additional file 3 (Suppl. Table 3. Univariate logistic regression analysis relatedto LVD and SVD). The 89+104 c>t was significantly associated with LVD patients in multivariate analysis, including age, sex, TG, TC (HDL-C and LDL-C), FIB, hypertension, diabetes mellitus type 2 and smoking. The event of LVD was significantly reduced in tt compared with cc individuals (odds ratio of tt, 0.261; 95% CI, 0.130 to 0.527; P < 0.0001). Conversely, this translated to an approximate 4-fold increased risk (odds ratio, 3.825; 95% CI, 1.897 to 7.713; P < 0.0001) for cc individuals relative to tt. However, 89+104 c>t was not significantly associated with LVD patients in male subgroup and ≤ 60 years subgroup. T allele of 89+104 c>t was also associated with SVD stroke (odds ratio of t allele, 0.278; 95% CI, 0.156 to 0.495; P < 0.0001), especially in female subgroup (odds ratio of t allele, 0.250; 95% CI, 0.102 to 0.612; P = 0.002). The LDL-C level of t allele carriers was lower than c/c homozygotes. In t allele carriers, hypertension and hypercholesterolemia (Total Cholesterol ≥ 240 mg/dl or 6.22 mmol/L) [37] were less common compared with c/c homozygotes.

**Table 2:** Multivariate logistic regression analysis of *Ngb *polymorphisms related to LVD and SVD

		**LVD**		**SVD**	
			
		Odds Ratio (95%CI) *	P*	Odds Ratio (95%CI) *	P*
89+104 c>t	cc	reference		reference	
	ct	0.395 (0.219–0.711)	0.002	0.289(0.157–0.533)	<0.0001
	tt	0.261(0.130–0.527)	<0.0001	0.256(0.127–0.515)	<0.0001
	c				
	t	0.351(0.199–0.617)	<0.0001	0.278(0.156–0.495)	<0.0001
322–110 (6a)>5a	6a6a	reference		reference	
	6a5a	1.164(0.665–2.037)	0.594	0.746(0.409–1.361)	0.340
	5a5a	1.292(0.657–2.543)	0.458	1.336(0.662–2.697)	0.418
	6a				
	5a	1.199(0.702–2.048)	0.506	0.894(0.505–1.582)	0.701

* Hypertension, DM 2, Smoking, TG level, HDL-C and LDL-C level have been adjusted.

**Table 3:** Multivariate logistic regression analysis of 89+104 c>t related to female and >60 years subgroups

89+104 c>t	**Female LVD vs control**		**Female SVD vs control**		**>60 years LVD vs control**	
	
	Odds Ratio (95%CI)*	P*	Odds Ratio (95%CI)*	P*	Odds Ratio(95%CI) **	P**
cc	reference		reference		reference	
ct	0.634(0.231–1.740)	0.376	0.444(0.163–1.204)	0.111	0.242(0.104–0.563)	0.001
tt	0.062(0.017–0.230)	<0.0001	0.128(0.044–0.371)	<0.0001	0.219(0.086–0.556)	0.001
c	reference		reference		reference	
t	0.283(0.112–0.716)	0.008	0.250(0.102–0.612)	0.002	0.233(0.104–0.521)	<0.0001

* Age, Hypertension, DM 2, Smoking, TG level, HDL-C and LDL-C level have been adjusted.

** Sex, Hypertension, DM 2, Smoking, TG level, HDL-C and LDL-C level have been adjusted.

### Association with Ngb Haplotypes

Analysis of the two above genotypes resulted in 4 possible haplotypes and the frequencies were given in Table 4. The haplotype 89+104c/322-110(5a) (c-5a) was more frequent in both LVD and SVD groups while the haplotype 89+104t/322-110(6a) (t-6a) was more frequent in controls. The c-5a occurred in 33.7% of the LVD cases versus 21.7% of controls, yielding an odds ratio (OR) of 1.838 (95% CI, 1.318 to 2.563; P < 0.0001). And the effect of c-5a on SVD risk was in the same direction as that observed in LVD strokes (OR1.997, 95% CI, 1.384 to 2.880; P < 0.0001). Conversely, the t-6a occurred in 32.8% of controls versus 20.8% of LVD and 23.3% of SVD, with the ORs of 0.540 (95% CI, 0.389 to 0.750; P < 0.0001) and 0.623 (95% CI, 0.431 to 0.900; P = 0.011) respectively.

**Table 4:** Association study with *Ngb *haplotypes in LVD and SVD patients

Haplotype Poly1/Poly2	LVD vs Control				SVD vs Control			
	
	LVD n (%)	Control n (%)	OR (95%CI)	P	SVD n (%)	Control n (%)	OR (95%CI)	P
c-6a	131.91(29.8)	62.48(19.8)	1.726(1.224–2.434)	0.002	64.59(24.1)	62.48(19.8)	1.288(0.869–1.910)	0.201
c-5a	149.09(33.7)	68.52(21.7)	1.838(1.318–2.563)	<0.0001	95.42(35.6)	68.52(21.7)	1.997(1.384–2.880)	<0.0001
t-6a	92.09(20.8)	103.53(32.8)	0.540(0.389–0.750)	<0.0001	62.42(23.3)	103.53(32.8)	0.623(0.431–0.900)	0.011
t-5a	68.91(15.6)	81.47(25.8)	0.532(0.371–-0.762)	0.001	45.58(17.0)	81.47(25.8)	0.590(0.393–0.886)	0.010
Global χ^2^				36.407				20.966
Fisher P				<0.0001				<0.0001

Poly1: 89+104 c>t; Poly2: 322-110 (6a)>5a

## Discussion

For *Ngb *is hypoxia/ischemia-upregulated and shows the neuroprotective potential during hypoxic/ischemic stress, the *Ngb *polymorphisms may be one of the candidates for being a genetic factor associated with ischemic stoke. In the present study, we have investigated the association of *Ngb *polymorphisms with ischemic stoke in the Southern Chinese Han population.

It has been suggested that the pathogenesis in ischemic stroke may be heterogeneous, differing between patients with LVD stroke, SVD stroke or CE stroke. Therefore, the studies of genetic factors for ischemic stroke should subdivide according to the different clinical or etiological subtypes to avoid false positive or negative results [38,39]. TOAST criteria were used to determine stroke etiology, because they are thought to be the best available clinical criteria to separate different stroke etiologies, although they present an imperfect relationship to the underlying inherited disease mechanism.

The cDNA sequence of *Ngb *is highly conservative. No mutations or polymorphisms within cDNA sequence were found in subjects analyzed. However, two polymorphisms 89+104c>t and 322-110(6a)>5a were detected in exon flanking sequence (95–303 bp). Other polymorphisms like rs28988618, rs101400032, rs7149300 and rs10133981 were not identified in the present study.

According to the SNP on NCBI Reference Assembly, the t carrier frequency of 89+104c>t was 50%, 21.4%, 66.7% in European, African and Asian populations respectively [40]. All the above data came from small sample ranged from 14 to 46 subjects. The t carrier frequency of the control group in the present study was 58.5%. The 6a allele frequency of 322-110 (6a)>5a was lower in the control gruop of our study (52.5% vs 75%, 87.5% and 63% in European, African and Asian populations) [41].

The significant differences in 89+104c>t genotype distribution and allele frequency were found between LVD patients or SVD patients and controls, which indicated this polymorphism might be correlated with LVD and SVD. T allele frequencies of LVD patients (36.4%) and SVD patients (40.3%) were significantly lower than that of the controls (58.5%). Conditional logistic regression analysis showed that the presence of at least one t allele in 89+104c>t were probably potential independent protective factor, while hypertension was independent risk factors for both LVD and SVD stroke. DM2, TG and LDL-C were independent risk factors for LVD stroke but not for SVD stroke, while HDL-C was protective factor for LVD stroke.

As we all known, confounding bias should conceal or exaggerate the association between exposure factor and the disease, thus resulted in the false negative or false positive. Age-bias and gender-bias are important considerations in case-control study for cerebrovascular disease. Data was analyzed by cluster stratification analysis to exclude the interference of these confusion factors in this study. T allele of 89+104c>t might be one of the independent protect factors for LVD in females and people over 60 years. The risk of LVD was further decreased in female t allele carriers but not in males. Age stratified analysis suggested that t allele could reduce the LVD risk on people over 60 years, but its protective effect on people under 60 years was not significant. We also confirmed that t allele might be a new protective factor for female SVD. Since t allele was much more frequent in females than in males, and t allele was significant protective especially for females, we presumed that there should be some correlations between *Ngb *protective effect and female hormones, which remains to be confirmed by further studies.

The genotype distributions and allele frequencies of *Ngb *322-110 (6a)>(5a) were similar between cases and controls, which suggesting this site had no obvious correlation with LVD or SVD. However, the haplotype 89+104c/322-110(5a) showed a higher distribution frequency in cases than in controls (OR = 1.838 for LVD, 1.997 for SVD separately), indicating significant association with LVD and SVD. Gradual increase of statistical power with the inclusion of two polymorphisms supports the validity of our conclusion that the *Ngb *is a susceptibility locus for ischemic stroke.

Our data also showed that *Ngb *89+104 t allele carriers have a lower LDL-C level (P = 0.003), less frequent hypercholesterolemia (P = 0.008) and hypertension (P < 0.0001) compared with c/c homozygotes. It is possible that *Ngb *plays its role against ischemic injury related to blood lipid metabolism or blood pressure regulation, which probably find new way to investigation of *Ngb *protective mechanism. When the sex was analyzed separately, we found that the carrier of t allele have low LDL-C (P = 0.002), slightly high HDL-C level (P = 0.012) and low frequent hypercholesterolemia (P = 0.003) and hypertension (P < 0.0001) in male subgroup. We concluded that the 89+104c>t polymorphism show significant association with plasma lipids level (LDL-C and HDL-C), hypercholesterolemia and hypertension especially in Chinese male.

As we all known, exonic polymorphisms have a potential to change amino acid coding sequence. The current model of pre-mRNA splicing is based on the recognition of four canonical intronic motifs (5' splice site, branchpoint sequence, polypyrimidine (PY) tract and 3' splice site), however, it has become clear that the four canonical splice elements do not contain adequate sequence information to ensure accurate splicing. A few families of motifs that are over-represented upstream of weak PY tracts were identified and suggested as intronic splicing enhancers (ISEs) that appear to compensate for a weakened canonical pre-mRNA splicing motif in both short and long human introns [42-44]. Some intronic SNP, for example, the intronic prothrombin 19911A>G is itself functional and changes splicing efficiency by altering a known functional pentamer motif CAGGG [45]. The intronic 89+104 c>t SNP could probably be assumed to influence the splicing efficiency of *Ngb *by toggling between CCGGG and CTGGG, which is one of the above motifs. *Ngb*'s structure shows a peculiar internal cavity of very large size. Binding of heme ligands is associated to a conformational change involving the heme that "slides" into the pre-existing cavity and makes the sixth coordination position available [46]. So the spatial conformation of *Ngb *is especial important. We presumed that whether this intronic SNP influenced nucleotide splicing and formed different spliceosomes which might change the classical α helical and three-over-three sandwich structure of *Ngb*, and then resulted in the interference of protein folding, instead of changing the amino acid coding sequence. Of course, additional experimental evidence will need to be obtained to verify the functional significance of the SNP identified by our study.

Some potential biases may still have influenced our final results. Prevalence of risk factors and stroke subtypes differ between hospitalized and community patients with ischemic stroke. This case-control study was mainly hospital-based, with comprehensive and accurate data, but there would be inevitable selection bias compared with community-based study. The lack of integrity data for carotid ultrasound examination in controls may be another weakness of this study, thus we have to reject carotid internal media thickness data in our study.

## Conclusion

In conclusion, t allele of 89+104c>t (rs4903565) might have a protective effect against LVD and SVD in the Southern Chinese Han population, especially for female or people over 60 years. *Ngb *322-110(6a)>5a polymorphism showed association with neither LVD nor SVD subtype. However, there was a "hitchhiking" effect for ischemic stroke was observed for the 89+104 t/322-110 (6a) genotype combination especially for LVD. To our knowledge, this is the first study on correlation between *Ngb *polymorphisms and ischemic stroke has been investigated. These results need to be further confirmed by some multicenter case-control study or prospective study in different ethnic populations.

## Abbreviations

*Ngb*: *Neuroglobin*; *Mb*: *myoglobin*; *Hb*: *hemoglobin*; LVD: stroke caused by large-artery atherosclerosis; SVD: stroke caused by small-vessel occlusion; BMI: body mass index; DM2: diabetes mellitus type 2; LDL-C: low density lipoprotein cholesterol; HDL-C: high density lipoprotein cholesterol; TC: total cholesterol; TG: triglycerides; FIB: fibrinogen; PCR: polymerase chain reaction; RFLP: Restriction fragment length polymorphism; PY: polypyrimidine; ISEs: intronic splicing enhancers.

## Competing interests

*Neuroglobin*, a newly discovered member of the globin superfamily, is expressed predominantly in brain neurons, and modulate hypoxic-ischemic insults. Although the mechanisms underlying *Ngb*-mediated neuronal protection are still unclear, *Ngb *appears to be one of the candidate protective factors for ischemic stroke. This is the first case-control study to clarify the association of *Ngb *polymorphisms with ischemic stroke in the world. And we found that *Ngb *polymorphism 89+104 t had protective effects on ischemic stroke, including large-artery atherosclerosis (LVD) and small-vessel occlusion (SVD) strokes in the Southern Chinese Han population. Meanwhile, a "hitchhiking" effect was observed for the 89+104 t/322-110 (6a) genotype combination especially for LVD.

## Authors' contributions

YL carried out the molecular genetic studies, participated in the analysis of the data and drafted the manuscript. LF and XHX collected demographic data and risk factor profiles of subjects and participated in analysis and interpretation of data. SXMR analyzed the clinical data of all subjects. NW participated in the design of the study and the acquisition of data. ZYW conceived of the study, and participated in its design and coordination, and revising the manuscript critically for important intellectual content. All authors have read and approved the final version of the manuscript.

## Pre-publication history

The pre-publication history for this paper can be accessed here:



## Supplementary Material

Additional file 1 **Supplementary Table 1**. Primers of Ngb and conditions of PCR. All primers used for PCR are illustrated in this PDF file. Click here for file

Additional file 2 **Supplementary Table 2**. Clinical and biochemical characteristics of three groups. Demographic data and risk factor profiles of patients and controls are presented in this PDF file. Concerning the gender, BMI, alcohol intake and smoking, there were no significant differences between cases and controls. However, some risk factors, such as hypertension, diabetes, LDL-C, HDL-C, TC and TG levels did differ between them. Click here for file

Additional file 3 **Supplementary Table 3**. Univariate logistic regression analysis related to LVD and SVD. This is a PDF file. Click here for file
