# Supplementary material for: Association between Ngb polymorphisms and ischemic stroke in the Southern Chinese Han population
Source: BMC Med Genet. 2008 Dec 16;9:110. doi: 10.1186/1471-2350-9-110 (PMC2639551; doi:10.1186/1471-2350-9-110)
Supplement: Additional file 1 — Supplementary Table 1. Primers of Ngb and conditions of PCR. All primers used for PCR are illustrated in this PDF file. [file 1471-2350-9-110-S1.pdf]

Supplementary Table 1. Primers of *Ngb* and conditions of PCR

Primers		Oligonucleotide of primers	Size of PCR product	Annealing temperature
Exon1	F	5’-TGTCTCCACCTACGACTG-3’	430 bp	65℃
	R	5’-CTCCTCTGGCGCACATCT-3’		
Exon2	F	5’-ACTCCAGGTTTCCTGTTTGG-3’	597bp	55℃
	R	5’-GGAAGAGGCTGATTCTGATG-3’		
Exon3	F	5’-TCTCTTGCTTTGGGACCCTC-3’	399bp	56℃
	R	5’-CTTGGAAGCTCAGAGAGGTC-3’		
Exon4	F	5’-GGGAAAGGTAGACACAATGTG-3’	508bp	60℃
	R	5’-GAAAAGCCACTCCTTCTGCAG-3’		
KpnI*Ngb*	F	5’-CGAAGGAGGGAAAACTGGCCGGTAC-3’	284bp	61℃
	R	5’-TGCAGTCGCTACCTAAGGAAGACGC-3’		
