# Supplementary material for: Association between Ngb polymorphisms and ischemic stroke in the Southern Chinese Han population
Source: BMC Med Genet. 2008 Dec 16;9:110. doi: 10.1186/1471-2350-9-110 (PMC2639551; doi:10.1186/1471-2350-9-110)
Supplement: Additional file 2 — Supplementary Table 2. Clinical and biochemical characteristics of three groups. Demographic data and risk factor profiles of patients and controls are presented in this PDF file. Concerning the gender, BMI, alcohol intake and smoking, there were no significant differences between cases and controls. However, some risk factors, such as hypertension, diabetes, LDL-C, HDL-C, TC and TG levels did differ between them. [file 1471-2350-9-110-S2.pdf]

Supplementary Table 2. Clinical and biochemical characteristics of three groups

	Control	LVD		SVD	
	n=158	n=221	P	n=134	P
Age (years)	65.08±10.44	66.61±10.47	0.670	66.96±7.62	0.086
Male/Female	103/55	160/61	0.143	76/58	0.149
BMI(kg/m^2)	23.30±2.20	24.40±2.40	0.845	24.50±3.20	0.127
Hypertension (%)	41.14	67.87	<0.0001	61.19	0.001
DM2 (%)	17.72	31.67	0.003	25.37	0.117
Smoking (%)	7.59	14.93	0.036	14.17	0.86
Alcohol intake(%)	2.63	4.66	0.500	3.05	1.000
HDL-C(mmol/L)	2.73±1.13	2.17±1.14	<0.0001	2.68±1.02	0.026
LDL-C(mmol/L)	1.42±0.64	1.98±1.10	<0.0001	1.46±0.80	0.007
TC(mmol/L)	4.50±0.76	4.72±0.95	0.002	4.74±0.92	0.010
TG(mmol/L)	1.48±0.62	1.75±1.10	<0.0001	1.65±0.98	0.043
FIB(g/L)	3.30±0.76	4.04±1.24	<0.0001	3.75±1.04	0.005

LVD: stroke caused by large-artery atherosclerosis; SVD: stroke caused by small-vessel occlusion; BMI: body mass index; DM2: diabetes mellitus type 2; LDL-C: low density lipoprotein cholesterol; HDL-C: high density lipoprotein cholesterol; TC: total cholesterol; TG: triglycerides; FIB: fibrinogen.
