# Supplementary material for: Association between Ngb polymorphisms and ischemic stroke in the Southern Chinese Han population
Source: BMC Med Genet. 2008 Dec 16;9:110. doi: 10.1186/1471-2350-9-110 (PMC2639551; doi:10.1186/1471-2350-9-110)
Supplement: Additional file 3 — Supplementary Table 3. Univariate logistic regression analysis related to LVD and SVD. This is a PDF file. [file 1471-2350-9-110-S3.pdf]

Supplementary Table 3. Univariate logistic regression analysis related to LVD and SVD

		LVD		SVD	
		Odds Ratio (95%CI)	P	Odds Ratio (95%CI)	P
Hypertension		3.023 (1.977-4.621)	<0.0001	2.256(1.410-3.611)	0.001
DM2		2.152 (1.309-3.538)	0.003	1.579(0.898-2.775)	0.113
TG		1.444 (1.108-1.883)	0.007	1.303(0.961-1.765)	0.088
HDL-C		0.590(0.479-0.728)	<0.0001	0.947(0.738-1.215)	0.668
LDL-C		1.954 (1.520-2.513)	<0.0001	1.068(0.775-1.471)	0.689
Smoking		2.136 (1.066-4.280)	0.032	2.010(0.937-4.311)	0.073
89+104 c>t	cc	reference		reference	
	ct	0.285(0.166-0.490)	<0.0001	0.291(0.161-0.529)	<0.0001
	tt	0.160(0.085-0.302)	<0.0001	0.230(0.116-0.455)	<0.0001
322-110 (6a)>5a	6a6a	reference		reference	
	6a5a	0.987(0.600-1.622)	0.958	0.873 (0.494-1.540)	0.638
	5a5a	1.187(0.645-2.184)	0.582	1.533 (0.789-2.979)	0.207
